# Tumour necrosis factor-α mediates blood—brain barrier damage in HIV-1 infection of the central nervous system

**DOI:** 10.1155/S0962935192000292

**Published:** 1992

**Authors:** M. K. Sharief, M. Ciardi, E. J. Thompson, F. Sorice, F. Rossi, V. Vullo, A. Cirelli

**Affiliations:** 1 Department of Neurochemistry Institute of Neurology The National Hospital for Neurology and Neurosurgery Queen Square London WC1N 3BG UK; 2 Institute of Infectious Diseases University of Rome “La Sapienza” Elena 331 Rome 00161 Italy; 3 Department of Infectious Diseases University of Pisa Pisa 2013 Italy

## Abstract

The pathogenesis of brain inflammation and damage by human immunodeficiency virus (HIV) infection is unclear. Because blood–brain barrier damage and impaired cerebral perfusion are common features of HIV-1 infection, we evaluated the role of tumour necrosis factor-α (TNF-α) and interleukin-1β (IL-1β) in mediating disruption of the blood–brain barrier. Levels of TNF-α were more elevated in cerebrospinal fluid (CSF) than in serum of HIV-1 infected patients and were mainly detected in those patients who had neurologic involvement. Intrathecal TNF-α levels correlated with signs of blood–brain barrier damage, manifested by high CSF to serum albumin quotient, and with the degree of barrier impairment. In contrast, intrathecal IL-1β levels did not correlate with blood-brain barrier damage in HIV-1 infected patients. TNF-α seems to be related to active neural inflammation and to blood–brain barrier damage. The proinflammatory effects of TNF-α in the nervous system are dissociated from those of IL-1β.


Mediators of Inflammation, 1, 191–196 (1992)

### Tumour necrosis factor-α mediates blood—brain barrier damage in HIV-1 infection of the central nervous system

M. K. Sharief,[1,CA] M. Ciardi,[1]

E. J. Thompson,[1] F. Sorice,[2] F. Rossi,[2]

V. Vullo[2] and A. Cirelli[3]

[1]Department of Neurochemistry, Institute of Neurology, The National Hospital for Neurology and Neurosurgery, Queen Square, London WC1N 3BG, UK;
[2]Institute of Infectious Diseases, University of Rome "La Sapienza", Elena 331, 00161 Rome, Italy;
[3]Department of Infectious Diseases, University of Pisa, 2013 Pisa, Italy

[CA] Corresponding Author

**THE pathogenesis of brain inflammation and damage by human immunodeficiency virus (HIV) infection is unclear. Because blood–brain barrier damage and impaired cerebral perfusion are common features of HIV-1 infection, we evaluated the role of tumour necrosis factor-α (TNF-α) and interleukin-1β (IL-1β) in mediating disruption of the blood–brain barrier. Levels of TNF-α were more elevated in cerebrospinal fluid (CSF) than in serum of HIV-1 infected patients and were mainly detected in those patients who had neurologic involvement. Intrathecal TNF-α levels correlated with signs of blood–brain barrier damage, manifested by high CSF to serum albumin quotient, and with the degree of barrier impairment. In contrast, intrathecal IL-1β levels did not correlate with blood–brain barrier damage in HIV-1 infected patients. TNF-α seems to be related to active neural inflammation and to blood–brain barrier damage. The proinflammatory effects of TNF-α in the nervous system are dissociated from those of IL-1β.**

**Key words:** Blood–brain barrier, Brain inflammation, HIV-1 infection, Interleukin-1, Tumour necrosis factor

### Introduction

Neurological complications are a very significant feature of HIV infection at all stages ranging from HIV seropositivity through to acquired immune deficiency syndrome (AIDS)-related complex and the full-blown AIDS.[1] About 10% of patients with HIV infection present neurologically,[2] while approximately 70% of patients with AIDS have some evidence of neurologic involvement and this figure may extend to 80% if pathological data are also taken into consideration.[1]

One of the most important neurologic complications is an HIV encephalitis, also known as AIDS—dementia complex, which is caused by a direct HIV infection within the brain.[3] However, the precise pathogenesis of brain inflammation and injury has not been clearly defined and although HIV has been demonstrated within macrophages and multinucleate giant cells,[4] its localization within glial cells and neurons has not been demonstrated convincingly. The amount of virus detected in some brain lesions is not proportionate to the degree of pathologic damage, and some brain regions with significant damage (e.g. spinal cord) contain little or no HIV.[5] Similarly, humorally mediated immune mechanisms are not significantly involved in the pathogenesis of CNS inflammation or injury.[5]

It is therefore likely that indirect mechanisms, such as the release of cytokines, may play an important role in mediating brain inflammation in HIV infection. Indeed there is now increasing evidence that tumour necrosis factor-α (TNF-α), which is a central mediator of inflammation, plays a crucial role in the development of AIDS.[6] TNF-α enhances the replication of HIV and induces the expression of a wide array of inflammatory cytokines. Moreover, TNF-α selectively kills HIV-infected cells, probably through a direct cytotoxic effect, and is currently implicated in the pathogenesis of most clinical and pathologic features of AIDS.[6]

Another important pathologic feature that may contribute to brain damage in HIV infection is the impairment of the blood–brain barrier. The detection of viral antigen in cerebral endothelial cells[4] has fuelled speculation that viral entry into the central nervous system (CNS) may be through the blood–brain barrier.[7] It follows that HIV infection of endothelial cells may alter the integrity of the blood–brain barrier, thereby augmenting neurologic dysfunction.[6] Indeed several investigators have reported a significant impairment of the blood–brain barrier in HIV brain inflammation, which ranged from 27% in the early infection[8] to 79% in more advanced stages.[9] In addition, changes

© 1992 Rapid Communications of Oxford Ltd in cerebral perfusion occur early in the course of HIV infection,[10] and HIV seropositive patients appear to be at increased risk of cerebral ischaemia and infarction.[11,12] However, the nature and mechanisms involved in HIV-related damage to cerebral endothelial cells and the blood–brain barrier are presently not well understood.

There is considerable evidence that TNF-α induces inflammatory changes on human cerebral endothelial cells. Effects of TNF-α that are relevant to HIV infection include modulation of endothelial cell functions,[13,14] resulting in endothelial damage, and an increase in vascular endothelial permeability leading to vascular leak syndrome[15] and impaired cerebral perfusion. It is therefore likely that TNF-α induced cerebrovascular disturbance may lead to brain damage in HIV infected patients. Some of the proinflammatory effects of TNF-α were reported to be influenced by interleukin-1β (IL-1β).[16] Thus, the aim of this study was to analyse the *in vivo* relationship of TNF-α and IL-1β to impairment of blood–brain barriers in patients with HIV infections.

### Patients and Methods

*Patients:* Paired CSF and serum samples were obtained from 31 HIV type-1 (HIV-1) seropositive patients (25 males, six females). Their ages ranged from 18 to 43 years (median age of 29.1 years). Nineteen patients were intravenous drug abusers, two had a drug-abusing partner, seven were homosexual (including one who was also a drug abuser), two were haemophiliacs, and one patient had received a blood transfusion.

Patients were classified according to the Walter Reed classification[17] (Table 1). Group IV C1 patients who had opportunistic infections of CNS included cerebral toxoplasmosis (five patients), cryptococcal meningitis (three patients) and tuberculous meningitis (two patients). Group IV C1 patients who had opportunistic infections outside the CNS included *Pneumocystis carinii* pneumonia (five patients) and refractory pharyngoesophageal candidiasis (four patients). Neurosyphilis, frequently associated with HIV infection, was ruled out in all patients by fluorescent treponemal antibody absorption and treponemal haemoagglutination tests. Cerebrospinal fluid was obtained by lumbar puncture and cells were separated by cytocentrifugation then all samples were filtered through a 0.45 μm disposable sterile filter (Millipore, Watford, UK) to remove contaminating particulate materials. Samples were frozen in aliquots at −70°C and thawed just before use. Repeated thawing and refreezing was avoided.

**Table 1.** Summary of the clinical features of 31 HIV-1 seropositive patients included in the study

| Group | No. of patients | Clinical features |
|---|---|---|
| Group III | 3 | Persistent generalized lymphadenopathy |
| Group IV | | |
| Subgroup A | 3 | Constitutional disease |
| Subgroup B | 4 | Neurological manifestations |
| Subgroup C1 | 10 | Secondary infections within the CNS |
| | 9 | Secondary infections outside the CNS |
| Subgroup D | 2 | Secondary cancers |

CNS denotes central nervous system.

*Controls:* Control CSF and serum samples were obtained from two main groups of HIV-1 seronegative patients. The first was the neurologic control group, which included 20 age- and sex-matched patients with various non-inflammatory neurologic diseases in whom blood–brain barrier damage was detected at presentation. They included six patients with meningioma, four with craniopharyngioma, three with intracranial arteriovenous malformation, three with cerebrovascular diseases, two with benign intracranial hypertension, and two with obstructive hydrocephalus. The second group was the disease control, which consisted of 16 patients with viral meningitis or meningoencephalitis.

*Assays:* All assays were performed in a blinded fashion on coded sterile samples. Levels of TNF-α in CSF and homologous serum samples were determined by a sandwich-type enzyme-linked immunosorbent assay (ELISA) described previously.[18] The ELISA had a coefficient of variation of 4.8% and a lower limit of detection of 2 pg/ml. A standard curve was run on each ELISA plate using recombinant human TNF-α in serial dilutions. A bioassay utilizing highly sensitive WEHI cells[19] was employed to verify results obtained by the ELISA. Levels of IL-1β in CSF and serum samples were also measured by a sensitive enzyme immunoassay.[20] In our hands the assay had a CV of 5.2% and a detection limit of 35 pg/ml. The period of storage of the test samples did not affect the assay results. Albumin concentrations in CSF and serum samples were measured by electroimmunoassay.[21]

*Evaluation of blood–brain barrier:* The term blood–brain barrier describes the overall exclusionary interfaces that include the epithelium of the choroid plexus, the endothelial cells of cerebral capillaries, and the layer of cells lining the arachnoid membrane.[22] The integrity of the blood–brain barrier was evaluated by calculating the CSF to serum albumin quotient[23] ($Q_{alb}$) which is the best chemical indicator of barrier damage.[23–25] It is noteworthy, however, that measurement of $Q_{alb}$ represents an approximation to blood–brain barrier breakdown as it commonly measures breakdown of blood–CSF barrier. The choroid plexus, in particular, has no significant blood–tissue barrier function.

*Statistics:* Non-parametric Wilcoxon sum rank and Pearson's correlation matrix tests were used, as appropriate, for statistical analysis. Relation between cytokine levels and the degree of barrier damage was studied by Kruskal–Wallis one-way analysis of variance. The distribution of TNF-α and IL-1β in the study population was evaluated by confidence intervals for non-parametric data.[26]

### Results

*Cytokine levels:* TNF-α was detected in 18 (58%) CSF and 15 (48%) serum samples from HIV-1 seropositive patients (Figure 1). TNF-α was absent in the serum of three HIV-1 seropositive patients who had high CSF TNF-α concentration (54 ± 14.5 pg/ml). The TNF-α concentration in CSF correlated with serum concentration ($r = 0.73$, $p < 0.001$). All the ten subgroup IV C1 patients with opportunistic CNS infections had high CSF TNF-α levels whereas only two of the nine IV C1 patients with opportunistic infections outside the CNS had high TNF-α in CSF ($p < 0.05$). Elevated CSF levels of TNF-α were also detected in a patient from group III, in four patients from group IV B, and a patient from group IV D.

TNF-α could not be detected in CSF samples of patients with viral meningitis and was detected in the serum of a patient with meningioma and in both CSF and serum samples from a further four

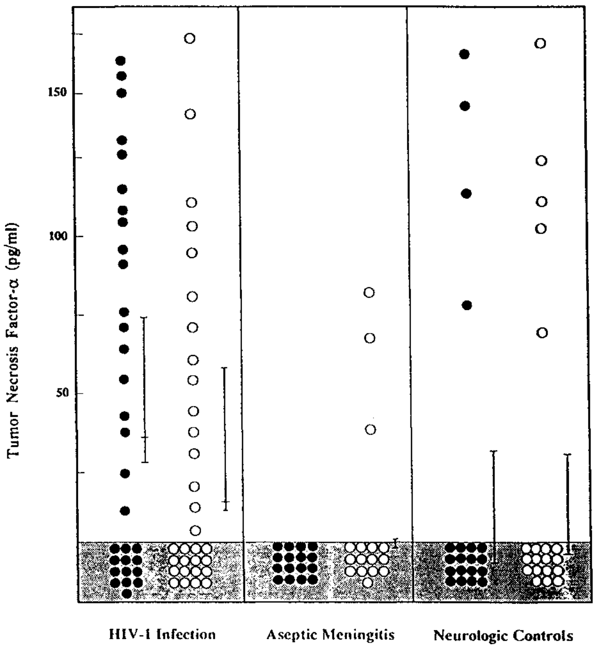

FIG. 1. Levels of TNF-α in CSF (●) and serum (○) in the study groups. Median values and 95% confidence intervals are shown. Shaded area represents the detection limit of the assay.

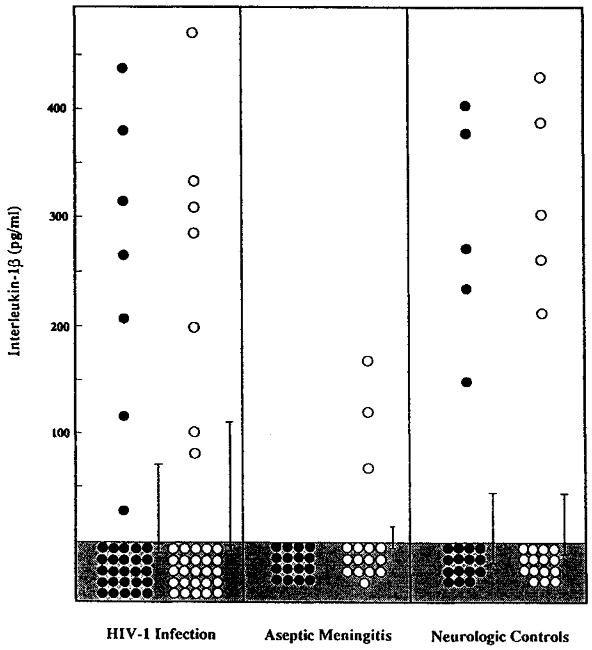

FIG. 2. Levels of IL-1β in CSF (●) and serum (○) in the study groups. Median values and 95% confidence intervals are shown. Shaded area represents the detection limit of the assay.

neurologic controls (Figure 1: two with stroke and two with craniopharyngioma).

Interleukin-1β was detected in seven (23%) CSF and serum samples from HIV-1 seropositive patients and in CSF and serum of five neurologic controls (Figure 2: two with stroke, one with meningioma and two with craniopharyngioma). CSF concentrations of IL-1β in HIV-1 seropositive patients did not correlate with CSF levels of TNF-α ($r = 0.32$, $p = 0.08$).

*Disruption of the blood–brain barrier:* We have already established[27] that the mean CSF albumin concentration in normal subjects was 198 mg/l (range 132 to 295) and the mean $Q_{alb}$ was 2.1 ± 1.6 × $10^3$ with a cut-off value of <6. Twenty-two (71%) patients with HIV infection had abnormally high $Q_{alb}$ suggestive of barrier impairment. The degree of disruption to the blood–brain barrier in the study population was graduated according to $Q_{alb}$ values, as described earlier,[24] which produced four separate groups of barrier condition (Table 2).

*Correlation of cytokine levels with barrier impairment:* Intrathecal amounts of TNF-α and IL-1β were determined by calculating the CSF to serum ratios of these cytokines to correct for passive transudation across damaged blood–brain barriers.[28,29] In HIV-1 seropositive patients who had detectable TNF-α levels, the CSF to serum ratio of TNF-α significantly correlated with $Q_{alb}$ (Figure 3) whereas HIV-1 seropositive patients who had no detectable TNF-α demonstrated no or only mild signs of blood–brain barrier damage (mean $Q_{alb}$ = 5.6 ±

**Table 2.** Condition of the blood—brain barrier in the study population

| Clinical groups (total number) | Degree of blood–brain barrier damage* | | | |
|---|---|---|---|---|
| | No damage | Mild | Moderate | Severe |
| HIV-1 seropositive patients (31) | 9 | 6 | 6 | 10 |
| Viral meningitis (16) | 7 | 4 | 4 | 1 |
| Neurologic controls (20) | 0 | 3 | 5 | 12 |

* According to cerebrospinal fluid/serum albumin quotient x $10^3$ where values <6 indicate no barrier damage, 6 to 10 = mild; 10.1 to 15 = moderate; and >15.1 to 30 = severe barrier damage.

$1.02 \times 10^{-3}$, $p < 0.01$). Moreover, the degree of barrier impairment in HIV-1 seropositive patients correlated with intrathecal TNF-α levels (Figure 4). In contrast, intrathecal levels of IL-1β in HIV-1 seropositive patients failed to correlate with $Q_{alb}$ ($r = 0.17$, $p = 0.12$) or with the degree of barrier damage (Figure 4).

### Discussion

In this study high levels of TNF-α were detected in CSF of HIV-1 seropositive patients who had neurologic involvement. In addition, TNF-α levels in CSF of these patients were higher than corresponding serum levels suggesting that TNF-α is released within the intrathecal compartment.[28,29] This finding is consistent with a previous report,[30] which detected an intrathecal production of TNF-α in HIV-1 seropositive patients with neurologic involvement. Gallo *et al.*,[31] however, failed to detect TNF-α in AIDS—dementia complex, even when extensive brain damage was detected. Such discrepancy may be due to variations in methodology or differences in patients selection.

The notion that TNF-α is released within the intrathecal compartment in HIV-1 infection of the CNS is supported by *in vitro* studies, which demonstrated that viral challenge of astrocytes induces TNF-α release.[32] Indeed CSF TNF-α levels were reported to be more elevated in patients with HIV-1 encephalopathy than in healthy HIV-1 seropositive subjects.[33] This was corroborated by our results, which showed that CSF levels of TNF-α were significantly higher in patients with HIV-1 encephalopathy or opportunistic CNS infections than in patients with systemic opportunistic infections without CNS involvement. Intrathecal production of TNF-α may also result from release

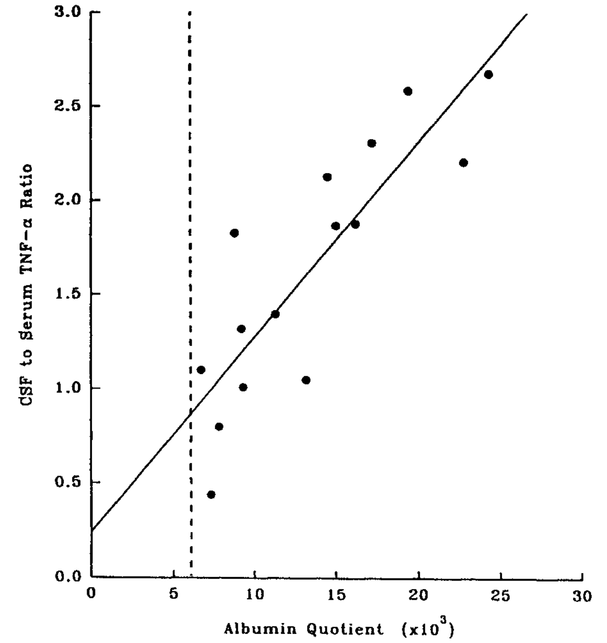

FIG. 3. Correlation of CSF to serum ratio of TNF-α with albumin quotient in 15 HIV-1 seropositive patients who had detectable TNF-α in CSF and serum. Vertical interrupted line represents the cut-off value of albumin quotient in normal subjects. $r = 0.86$, $p < 0.001$.

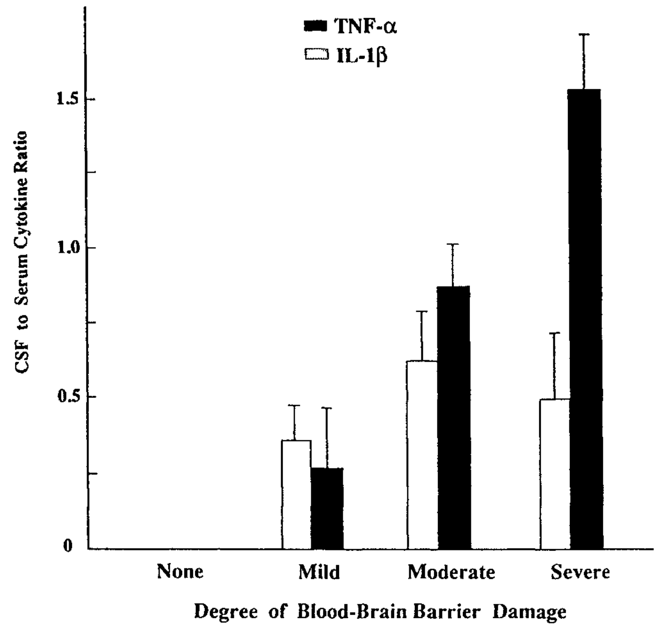

FIG. 4. Correlation of CSF to serum ratios of TNF-α and IL-1β with the degree of blood–brain barrier damage in 31 HIV-1 seropositive patients. Values represent means + SEM.

by macrophages, which are abundant in brain lesions, as well as by microglial cells.[34]

We detected a strong correlation between intrathecal levels of TNF-α and signs of disruption of the blood–brain barrier in HIV-1 seropositive patients with neurologic involvement. Moreover, TNF-α levels correlated with the degree of barrier impairment suggesting that this cytokine may be related to the pathogenesis of barrier damage in HIV-1 infection. Although our results are not sufficient to confirm a direct pathogenic effect of TNF-α on cerebral endothelial cells, a putative TNF-α-induced disruption of blood–brain barriers could result from several mechanisms. TNF-α induces morphologic and structural changes of endothelial cells through a direct toxic effect.[35] It also downregulates endothelial cell expression of thrombomodulin and causes enhanced procoagulant activity that promotes intravascular coagulation and capillary thrombosis.[13] In addition, leucocytes adherent to endothelial cells are stimulated by TNF-α to increase biosynthesis and release of reactive superoxide intermediates and arachidonic acid metabolites.[36] Further pathological studies are necessary to elucidate any direct pathological role of TNF-α in blood–brain barrier damage in HIV infection.

We detected no relationship between IL-1β level and blood–brain barrier damage while such damage correlated with TNF-α concentrations. Although it has been suggested that IL-1β may precipitate blood–brain barrier damage in experimental animals,[37] our data suggest that the effects of TNF-α on human cerebral endothelium can be dissociated from the presence of IL-1β. In support of our observation, Saukkonen *et al.*[38] reported a role of TNF-α in the generation of inflammation and tissue damage, while IL-1β failed to provoke a significant meningeal response in experimental animals. It must be emphasized, however, that endothelial damage should not be considered a result solely of overproduction of TNF-α, thereby ignoring the complex interactions between cytokines and other mediators such as prostaglandins and leukotrienes. Studies of these and other mediators should provide a further insight into the pathogenesis of brain inflammation in HIV infection.

In conclusion, our results provide a molecular basis for intrathecal inflammatory response in patients with HIV infection. They further extend the previously reported effects of TNF-α on the nervous system in HIV-1 infection and implicate TNF-α in the blood–brain barrier damage occurring in this disease. Analysis of the temporal relation between TNF-α-mediated barrier damage and other pro-inflammatory effects of TNF-α on neural tissues is necessary for a better understanding of HIV-mediated central nervous system damage.

### References

1. Kennedy PGE. Neurological complications of human immunodeficiency virus. *Postgrad Med J* 1988; **64**: 180–187.
2. Lavy RM, Bredesen DE, Rosenblum ML. Neurological manifestations of the acquired immunodeficiency syndrome (AIDS): experience at UCSF and review of the literature. *J Neurosurg* 1985; **62**: 475–495.
3. Shaw GM, Harper ME, Hahn BH, *et al.* HTLV-III infection in brains of children and adults with AIDS encephalopathy. *Science* 1985; **227**: 177–182.
4. Wiley CA, Schrier RD, Nelson JA, Lampert PW, Oldstone MBA. Cellular localization of human immunodeficiency virus infection within the brains of acquired immune deficiency syndrome patients. *Proc Natl Acad Sci USA* 1986; **83**: 7089–7093.
5. Lenhardt TM, Wiley CA. Absence of humorally mediated damage within the central nervous system of AIDS patients. *Neurology* 1989; **39**: 278–280.
6. Matsuyama T, Kobayashi N, Yamamoto N. Cytokines and HIV infection: is AIDS a tumour necrosis factor disease? *AIDS* 1991; **5**: 1405–1417.
7. Mizusawa H, Hirano A, Llena JF, Shintaku M. Cerebrovascular lesions in acquired immune deficiency syndrome. *Acta Neuropathol* 1988; **76**: 451–457.
8. Price RW, Brew B, Sidtis J, Rosenblum M, Scheck AC, Cleary P. The brain in AIDS: central nervous HIV-1 infection and AIDS dementia complex. *Science* 1988; **239**: 586–592.
9. Brew BJ, Bhalla RB, Fleisher M, *et al.* Cerebrospinal fluid $\beta_2$ microglobulin in patients infected with human immunodeficiency virus. *Neurology* 1989; **39**: 830–834.
10. Felgenhauer K, Luer W, Poser S. Chronic HIV encephalitis. *J Neuroimmunol* 1988; **20**: 141–144.
11. Schielke E, Tatsch K, Pfister HW, *et al.* Reduced cerebral blood flow in early stages of human immunodeficiency virus infection. *Arch Neurol* 1990; **47**: 1342–1345.
12. Engstrom JW, Lowenstein DH, Bredesen DE. Cerebral infarction and transient neurologic deficits associated with acquired immunodeficiency syndrome. *Am J Med* 1989; **86**: 528–532.
13. Nawroth PP, Stern DM. Modulation of endothelial cell hemostatic properties by tumor necrosis factor. *J Exp Med* 1986; **163**: 740–746.
14. Kahaleh MB, Smith EA, Soma Y, LeRoy EC. Tumor necrosis factor inhibits endothelial cell growth. *Clin Immunol Immunopathol* 1988; **49**: 261–272.
15. Tracey KJ, Wey H, Manogue KR, *et al.* Cachectin/tumor necrosis factor induces cachexia, anemia and inflammation. *J Exp Med* 1987; **167**: 1211–1227.
16. Pober JS, Gimbrone MA, Lapierre LA, *et al.* Overlapping patterns of activation of human endothelial cells by interleukin 1, tumour necrosis factor, and immune interferon. *J Immunol* 1986; **137**: 1893–1896.
17. Redfield RR, Wright DC, Tramont EC. The Walter Reed staging classification for HTLV-III/LAV infection. *N Engl J Med* 1986; **314**: 131–132.
18. Mitchie HR, Manogue KR, Spriggs DR, *et al.* Detection of circulating tumor necrosis factor after endotoxin administration. *N Engl J Med* 1988; **318**: 1481–1486.
19. Espevik T, Nissen-Meyer J. A highly sensitive cell line, WEHI 164 clone 13, for measuring cytotoxic factor/tumour necrosis factor from human monocytes. *J Immunol Meth* 1986; **95**: 99–105.
20. Ramilo O, Saez-Liorens X, Mertsola J, *et al.* Tumor necrosis factor α/cachectin and interleukin 1β initiate meningeal inflammation. *J Exp Med* 1990; **172**: 497–507.
21. Ganrot K, Laurell CB. Measurement of IgG and albumin content of cerebrospinal fluid, and its interpretation. *Clin Chem* 1974; **20**: 571–573.
22. Felgenhauer K. The blood–brain barrier redefined. *J Neurol* 1986; **233**: 193–194.
23. Tibbling G, Link H, Ohman S. Principles of albumin and IgG analyses in neurological diseases. I. Establishment of reference values. *Scand J Clin Lab Invest* 1977; **37**: 285–390.
24. Schliep G, Felgenhauer K. Serum—CSF protein gradients, the blood–brain barrier and the local immune response. *J Neurol* 1978; **218**: 77–96.
25. Reiber H. The discrimination between different blood–CSF barrier dysfunctions and inflammatory reactions of the CNS by a recent evaluation graph for the protein profile of cerebrospinal fluid. *J Neurol* 1980; **224**: 89–99.
26. Campbell MJ, Gardner MJ. Calculating confidence intervals for some non-parametric analysis. *Br Med J* 1988; **296**: 1454–1456.
27. Sharief MK, Keir G, Thompson EJ. Intrathecal synthesis of IgM in neurological diseases: a comparison between detection of oligoclonal bands and quantitative estimation. *J Neurol Sci* 1990; **96**: 131–142.
28. Sharief MK, Hentges R. Association between tumor necrosis factor-α and disease progression in patients with multiple sclerosis. *N Engl J Med* 1991; **325**: 467–472.
29. Sharief MK, Thompson EJ. *In vivo* relationship of tumor necrosis factor-alpha to blood–brain barrier damage in patients with active multiple sclerosis. *J Neuroimmunol* 1992 (in press).
30. Grimaldi LM, Martino GV, Franciotta DM, *et al.* Elevated alpha-tumor necrosis factor levels in spinal fluid from HIV-1 infected patients with central nervous system involvement. *Ann Neurol* 1991; **29**: 21–25.
31. Gallo P, Piccinno MG, Krzalic L, Tavolato B. Tumor necrosis factor alpha and neurological diseases. Failure in detecting TNF alpha in the cerebrospinal fluid from patients with multiple sclerosis, AIDS dementia complex, and brain tumours. *J Neuroimmunol* 1989; **23**: 41–44.
32. Lieberman AP, Pitha PM, Shin HS, Shin ML. Production of tumor necrosis factor and other cytokines by astrocytes stimulated with lipopolysaccharide or a neurotropic virus. *Proc Natl Acad Sci USA* 1989; **86**: 6348–6352.

33. Perez VL, Janssen R, Spira T, *et al.* Presence of tumor necrosis factor in the cerebrospinal fluid of AIDS patients with HIV encephalopathy. Montreal: 5th International Conference on AIDS 1989; WCP87 (abstract).
34. Robbins DS, Shivazi Y, Drysdale BE, Lieberman A, Shin HS, Shin ML. Production of cytotoxic factor for oligodendrocytes by stimulated astrocytes. *J Immunol* 1987; **139**: 2593.
35. Sato N, Goto T, Haranaka K, *et al.* Actions of tumor necrosis factor on cultured vascular endothelial cells: morphologic modulation, growth inhibition, and cytotoxicity. *J Natl Cancer Inst* 1986; **76**: 1113–1121.
36. Nathan CF. Neutrophil activation on biological surfaces: massive secretion of hydrogen peroxide in response to products of macrophages and lymphocytes. *J Clin Invest* 1987; **80**: 1550–1560.
37. Quagliarello VJ, Wispelwey B, Long WJ, Sceld WM (1991) Recombinant human interleukin-1 induces meningitis and blood–brain barrier injury in the rat. Characterization and comparison with tumor necrosis factor. *J Clin Invest* 1991; **87**: 1360–1366.
38. Saukkonen K, Sande S, Cioffe C, *et al.* The role of cytokines in the generation of inflammation and tissue damage in experimental Gram-positive meningitis. *J Exp Med* 1990; **171**: 439.

ACKNOWLEDGEMENT. Dr M. Ciardi is supported by a grant from Instituto Superiore di Sanità, Italy.

**Received 27 March 1992; accepted 31 March 1992**
